# Ischemia In Vivo Induces Cardiolipin Oxidation in Rat Kidney Mitochondria

**DOI:** 10.3390/biology11040541

**Published:** 2022-03-31

**Authors:** Arvydas Strazdauskas, Sonata Trumbeckaite, Valdas Jakstas, Justina Kamarauskaite, Liudas Ivanauskas, Rasa Baniene

**Affiliations:** 1 Laboratory of Biochemistry, Neuroscience Institute, Lithuanian University of Health Sciences, LT-50162 Kaunas, Lithuania; sonata.trumbeckaite@lsmuni.lt (S.T.); rasa.baniene@lsmuni.lt (R.B.); 2 Department of Biochemistry, Medical Academy, Lithuanian University of Health Sciences, LT-50161 Kaunas, Lithuania; 3 Department of Pharmacognosy, Medical Academy, Lithuanian University of Health Sciences, LT-50162 Kaunas, Lithuania; valdas.jakstas@lsmuni.lt (V.J.); justina.kamarauskaite@lsmu.lt (J.K.); 4 Laboratory of Biopharmaceutical Research, Institute of Pharmaceutical Technologies, Lithuanian University of Health Sciences, LT-50162 Kaunas, Lithuania; 5 Department of Analytical and Toxicological Chemistry, Medical Academy, Lithuanian University of Health Sciences, LT-50162 Kaunas, Lithuania; liudas.ivanauskas@lsmuni.lt

**Keywords:** cardiolipin, ischemia, oxidation, mitochondria, kidney, oxidative stress

## Abstract

**Simple Summary:**

Mitochondrial cardiolipin is a unique phospholipid that plays a vital role in ATP synthesis. It has been observed that ischemia/reperfusion causes damage to cardiolipins in the heart or brain tissues; however, very little data has been found regarding kidney cardiolipins and related damage after ischemia/reperfusion. Oxidative stress plays a key role during reperfusion. However, even during ischemia, cardiolipins may be oxidized. Therefore, our aim was to evaluate cardiolipin oxidation during renal ischemia in vivo. Renal ischemia in vivo was induced in male Wistar rats for a 30–60-min period; we then isolated kidney mitochondria, extracted the lipids and analyzed cardiolipin by applying chromatographic and mass spectrometric methods. The results showed that after even 30 min of in vivo ischemia, the amounts of the dominant species of cardiolipin decreased almost in half, and it further decreased when extending the ischemia time. Cardiolipin was oxidized with up to eight additional oxygen atoms, yielding eight different species with multiple isomeric forms. This shows that even after ischemia, cardiolipin levels are altered, and many cardiolipin oxidation products are produced, which may also potentially be modified into more harmful lipid signaling molecules that may induce more damage to mitochondria.

**Abstract:**

Cardiolipin is a mitochondrial phospholipid that plays a significant role in mitochondrial bioenergetics. Cardiolipin is oxidized under conditions like oxidative stress that occurs during ischemia/reperfusion; however, it is known that even during ischemia, many reactive oxygen species are generated. Our aim was to analyze the effect of in vivo ischemia on cardiolipin oxidation. Adult male Wistar rats were anesthetized; then, their abdomens were opened, and microvascular clips were placed on renal arteries for 30, 40 or 60 min, causing ischemia. After ischemia, kidneys were harvested, mitochondria were isolated, and lipids were extracted for chromatographic and mass spectrometric analysis of tetralinoleoyl cardiolipin and its oxidation products. Chromatographic and mass spectrometric analysis revealed a 47%, 68% and 74% decrease in tetralinoleoyl cardiolipin after 30 min, 40 min and 60 min of renal ischemia, respectively (*p* < 0.05). Eight different cardiolipin oxidation products with up to eight additional oxygens were identified in rat kidney mitochondria. A total of 40 min of ischemia caused an average of a 6.9-fold increase in all oxidized cardiolipin forms. We present evidence that renal ischemia in vivo alone induces tetralinoleoyl cardiolipin oxidation and depletion in rat kidney mitochondria.

## 1. Introduction

Cardiolipin (CL) is a unique phospholipid that is mostly found in mitochondrial membranes and bacterial plasma membranes [1]. In the mitochondria, most CL is located in the inner membrane (IMM), where it makes up about 20% of total IMM phospholipid content [2]. The structure of this phospholipid is dimeric and includes four fatty acyl chains, which give rise to a variety of fatty acid combinations and cardiolipin molecular species. Even though many CL species are found in mammalian tissues, there is one species, tetralinoleyl-CL, that is the most abundant [3,4]. For example, this CL species comprises approximately 80% of all cardiolipins in the hearts of humans, rodents, and bovines. It has been observed that linoleic acid is the most abundant fatty acid in the cardiolipins that are found in liver, kidney, muscle and spleen tissues [5,6]. This unique structure of cardiolipin gives it special properties and functions within mitochondria. CL participates in mitochondrial membrane morphology, bioenergetics, metabolite transport, mitophagy, immune response and apoptosis [7,8,9,10]. Perhaps one of the most important functions of cardiolipins is their interaction with the mitochondrial respiratory chain complexes—cardiolipin is essential for their stability and the optimal function of the oxidative phosphorylation system. Previous studies have shown that cardiolipin is necessary for the optimal functioning of complex I, complex II, complex III, cytochrome c and adenosine triphosphate (ATP) synthase [10,11,12,13,14,15]. It has also been demonstrated that cardiolipin is able to increase the efficiency of oxidative phosphorylation by at least 35% [16]. Thus, any damage to cardiolipin may cause mitochondrial dysfunction and further pathological cascades. Changes in cardiolipin structure and amounts have been observed during such pathologies as Barth syndrome, cardiovascular and neurodegenerative diseases, and myocardial ischemia/reperfusion [3,17,18,19,20]. 

Structural changes of cardiolipin can occur mainly during oxidative stress when cardiolipin is oxidized through the attack of reactive oxygen species (ROS). Mitochondria are considered a major source of ROS production; therefore, considering the location of cardiolipin as well as its high number of polyunsaturated fatty acids, CL becomes highly susceptible to oxidation [21,22]. Under physiological conditions, ROS production and scavenging are highly controlled; however, it has been observed that ROS production increases under various pathological conditions such as ischemia/reperfusion, aging and degenerative diseases [23]. It has been shown that myocardial ischemia/reperfusion causes significant loss of cardiolipin in rat heart mitochondria and reduced activity in I, III and IV electron transport chain complexes [11,12,13]. Short periods of ischemia might not alter the antioxidant system; however, prolonged ischemia (e.g., 60 min of heart ischemia) damages the electron transport chain and increases ROS generation even when 1,5% oxygen is left [23,24]. Increased ROS generation during ischemia can already induce the oxidation of polyunsaturated fatty acids in CL with the formation of lipid peroxides and cause further damage to mitochondria.

Much research has been done analyzing cardiolipin alterations after myocardial ischemia/reperfusion [18,19,25,26]. However, there is not enough information about the implications of kidney ischemia/reperfusion on cardiolipin in kidney mitochondria. As there are no data about cardiolipin alterations during kidney ischemia at all, the aim of this study was to evaluate qualitative and quantitative changes of cardiolipin in kidney mitochondria after warm in vivo ischemia. This study demonstrates that kidney ischemia in vivo causes peroxidation of CL. Several molecular species of cardiolipin containing up to eight additional oxygen atoms attached to polyunsaturated fatty acids were identified using the ultra performance liquid chromatography-mass spectrometry (UPLC-MS) method.

## 2. Materials and Methods

### 2.1. Animals and Experimental Ischemia In Vivo Model

The experimental procedures used in the present study were performed according to the Lithuanian Committee of Good Laboratory Animal Use Practice (No. G2-170, 2021-03-02). Adult male Wistar rats weighing 200–250 g were housed under standard laboratory conditions, maintained on a natural light and dark cycle, and had free access to food and water ad libitum. Animals were acclimatized to laboratory conditions before the experiment. Pentobarbital and ketamine were used to perform anesthesia. Vascular clips were placed over the rats’ renal arteries to induce renal ischemia (37 °C) in vivo. Ischemia was confirmed by observing the kidneys change colour from red to purple within ~2 min. At the end of ischemia (30 min, 40 min or 60 min), the clips were taken off, and the kidneys were removed and washed free of blood in a cold (0–4 °C) 0.9% KCl solution. After that, kidney tissue was used for the isolation of mitochondria. Sham-operated rats (control group) underwent identical surgical procedures except for applying vascular clips.

### 2.2. Isolation of Kidney Mitochondria

Kidney tissue was cut into small pieces and homogenized in an isolation medium containing 250 mM sucrose, 10 mM Tris HCl, and 1 mM ethylenediaminetetraacetic acid (EDTA) (pH 7.3). Cytosolic and mitochondrial fractions were separated by differential centrifugation (5 min at 750× *g* and 10 min at 10,000× *g*, two times), and the pellet was suspended in an isolation medium. The pellet was used for protein determination by the Biuret method, mitochondrial function determination, and phospholipid extraction. 

### 2.3. Determination of Mitochondrial Function

**Figure 1:**
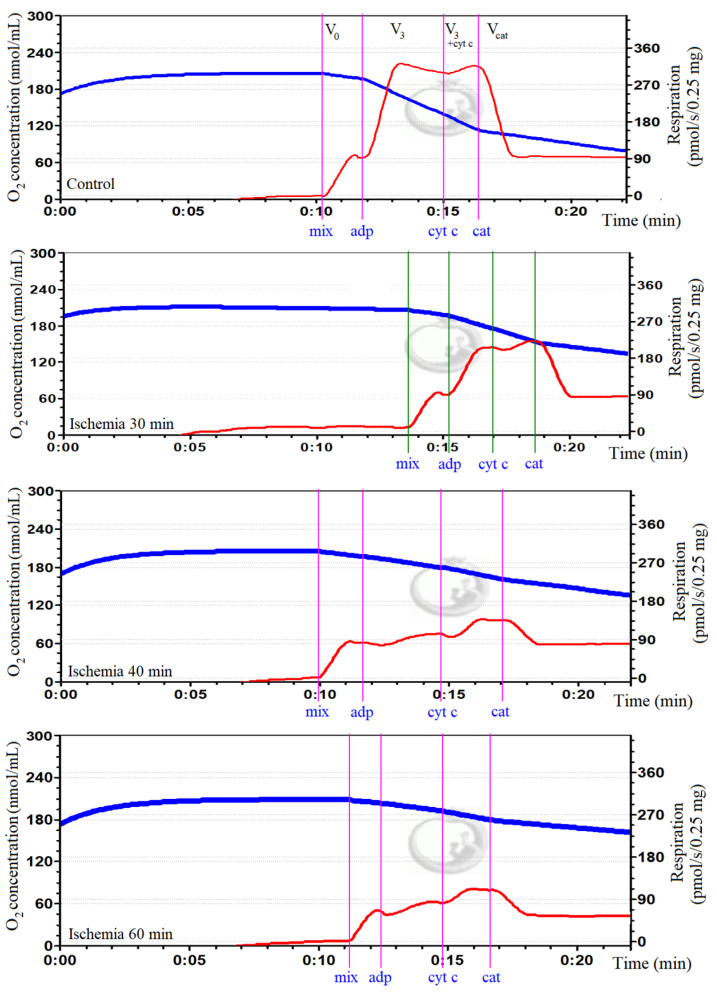
Oxygen consumption in kidney mitochondria in control and ischemia groups. In the curves, the blue trace represents oxygen concentration (nmol/mL), and the red trace represents oxygen flux (pmol/s/0.25 mg mitochondrial protein). V_0_: respiration rate in the presence of 0.5 mg/mL of mitochondria (mix) and substrates, 5 mM glutamate and 5 mM malate; V_3_: maximal respiration rate after adding 2 mM ADP; V_3+cytc_: respiration rate after adding 32 μM cytochrome c; V_cat_: respiration rate after adding 0.12 mM carboxyatractyloside. It was observed that mitochondrial respiration was diminished in all ischemia groups comparing to control.

Mitochondrial function was determined in order to confirm ischemic damage to mitochondria. This was done by measuring mitochondrial respiration (oxygen consumption) with the Oroboros-2k oxygraph system in 2 mL incubation medium (150 mM KCl, 10 mM Tris·Cl, 5 mM KH_2_PO_4_, 1 mM MgCl_2_ × 6H_2_O) at pH 7.3 and 37 °C. State II (V_0_) respiration was measured in the presence of 5 mM glutamate and 5 mM malate as complex I-dependent substrates. State III (V_3_) respiration was measured by adding 2 mM adenosine diphosphate (ADP). Mitochondrial outer membrane permeability was assessed by adding 32 μM cytochrome c (V_3+cytc_). The respiration was inhibited by adding 0.12 mM carboxyatractyloside (V_cat_), and changes in respiration rates were assessed, which indicated alterations in mitochondrial inner membrane permeability. Real-time data acquisition and data analysis were performed with Datlab 5 software (Oroboros Instruments, Innsbruck, Austria). Oxygen consumption rates were expressed as pmol/s/0.25 mg mitochondrial protein. Original curves of mitochondrial respiration in control and ischemic (30, 40, 60 min) groups are represented in Figure 1.

### 2.4. Extraction of Phospholipids from Kidney Mitochondria

Phospholipid extraction from kidney mitochondria was carried out using a modified Folch’s method [27]. A total of 500 μL of mitochondrial suspension was mixed with 1 mL of deionized water, 2 mL of methanol and 4 mL of chloroform (1:2:4 vol.). The mixture was centrifuged at 2000× *g* speed for five minutes at room temperature. The lower chloroform phase–containing lipids–was recovered and evaporated under a N_2_ stream in a glass vial. The remaining upper phase was mixed with an additional 2 mL of chloroform and centrifuged again. After that, the lower phase was recovered and added to the glass vial. This last step was repeated two times in order to recover as much lipid content as possible from the mixture. After concentration under a N_2_ stream, the pellet was resuspended in 100 µL of chloroform and 500 µL isopropanol (1:5 vol.) and filtered through a 45 µm syringe filter before the analysis.

### 2.5. Oxidation of Cardiolipin Standard 

Cardiolipin standard (cardiolipin sodium salt from bovine heart, ≥97% (TLC)), lyophilized powder, Sigma Aldrich, St. Louis, MO, USA) was oxidized using a method based on that reported by Kagan and Tyurina [28,29]. Cardiolipin standard was dissolved in phosphate buffer (K_2_HPO_4_ 50 mM, KH_2_PO_4_ 50 mM, double-distilled H_2_O; pH 7.4) and vortexed. The mixture was placed in an ultrasonic bath for 3 min to form lipid vesicles. The mixture was treated with cytochrome c (30 μM) and H_2_O_2_ (400 μM) for one hour at 37 °C in a water bath. H_2_O_2_ was added every 15 min (a total of four times) during the incubation period. Lipids were then extracted in chloroform/methanol solvent using a method described earlier. 

### 2.6. Separation and Evaluation of Cardiolipin 

Cardiolipin separation and analysis from our standard and mitochondrial samples was performed by ultra-effective liquid chromatography—mass spectrometry (UPLC-MS) using a Waters Acquity UPLC chromatograph coupled with a Xevo TQD (triple quadrupole) mass spectrometer with electrospray ionization (ESI). For UPLC analysis, an Acquity UPLC BEH C18 1.7 μm column was used. The method that was used was based on the reverse-phase ion-pair high-performance liquid chromatography-tandem mass spectrometry (HPLC-MS/MS) method that was described by Kim et al. [30]. Gradient elution used two eluents: eluent A (450 mL acetonitrile, 50 mL water, 2.5 mL triethylamine, 2.5 mL glacial acetic acid) and eluent B (450 mL isopropanol, 50 mL water, 2.5 mL triethylamine, 2.5 mL glacial acetic acid). The elution gradient is shown in Table 1.

The Xevo TQD quadrupole mass spectrometer (Waters Corporation, Millford, MA, USA) was operated in the negative scanning mode. Typical ESI conditions were as follows: the electrospray voltage was 2.50 kV, the source temperature was 150 °C, the desolvation temperature was 600 °C, the cone gas flow was 20 L/h, the desolvation gas flow was 1000 L/h, the MS collision energy was 35.00 eV, and the tandem mass spectrometry (MS/MS) collision energy was 22.00 eV. Scans were performed with negative ionization mode (ES-). The mass range was 200 to 1650, and the scan duration was 0.15 s.

### 2.7. Statistical Analysis

The quantitative data are presented as means ± standard error. Statistical analysis and graphic visualization were performed using the SigmaPlot v14.0 software package (Systat Software Inc., Chicago, IL, USA). For comparison of group means, one-way ANOVA was applied, followed by Dunn’s post hoc test for the pairwise multiple comparison procedure. Additionally, Student’s/Welch’s *t*-test was used for each pair mean comparison if the Shapiro–Wilk normality test was passed; Mann–Whitney Sum Rank test was used otherwise. *p* values < 0.05 were considered statistically significant. 

## 3. Results

### 3.1. Characterizing CL Standard by Reverse-Phase Ion Pair UPLC-MS/MS

**Figure 2:**
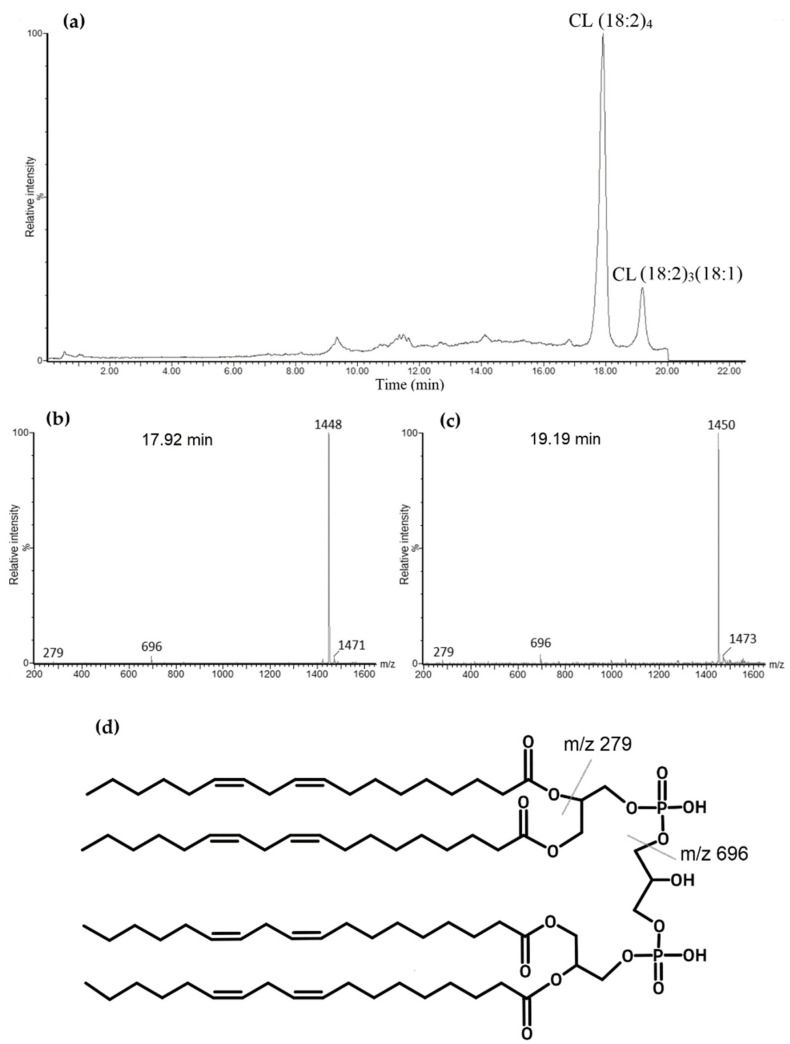
Total ion chromatogram of standard bovine cardiolipin (CL). (**a**). Two major chromatographic peaks are observed: the first one at 17.92 min with m/z of 1448 that corresponds with tetralinoleoyl CL—(**b**) corresponding mass spectrum; and another at 19.19 min with m/z of 1450 corresponding to CL (18:2)_3_(18:1)—(**c**) corresponding mass spectrum. (**d**) Tetralinoleoyl cardiolipin molecular structure with labeled fragments.

Cardiolipin is a diphosphatidylglycerol that has two phosphate groups that can be negatively charged. The components of elution buffers created an environment that allowed tetralinoleoyl CL to be a singly charged negative ion (M–H+) which was analyzed with negative mass spectrometry, ESI-. This resulted in two major chromatographic peaks at around 17.92 min and 19.19 min, which correspond to tetralinoleoyl CL (CL (18:2)_4_) and CL (18:2)_3_(18:1), respectively (Figure 2a). Both of these CL species were confirmed by analyzing their mass spectra. A 17.92 min peak was seen at m/z 1448, and a 19.19 min peak was seen at m/z 1450. Due to light fragmentation settings in MS/MS, the mass spectra showed one fragment at m/z 696, which corresponded to diacylglycerol phosphate (phosphatidic acid) with two linoleoyl fatty acids, as well as linoleic acid with m/z 279. These fragments were observed both at the 17.92 min and 19.19 min peaks (Figure 2b,c). One additional fragment of m/z 698 was found at 19.19 min, which corresponds to phosphatidic acid with C18:2 and C18:1 fatty acids. Additionally, mass spectra showed m/z 1471 and m/z 1473, which correspond to monosodiated tetralinoleoyl and (18:2)_3_(18:1) cardiolipins, respectively. It can be observed that these species have a shorter elution time compared to a previously published study with a similar methodology [28]. However, a clear separation is still maintained between CL (18:2)_4_ and CL (18:2)_3_(18:1). The tetralinoleoyl cardiolipin structure is also presented in Figure 2d.

### 3.2. Oxidation of Standard Cardiolipin and Identification of Oxidized Products

**Figure 3:**
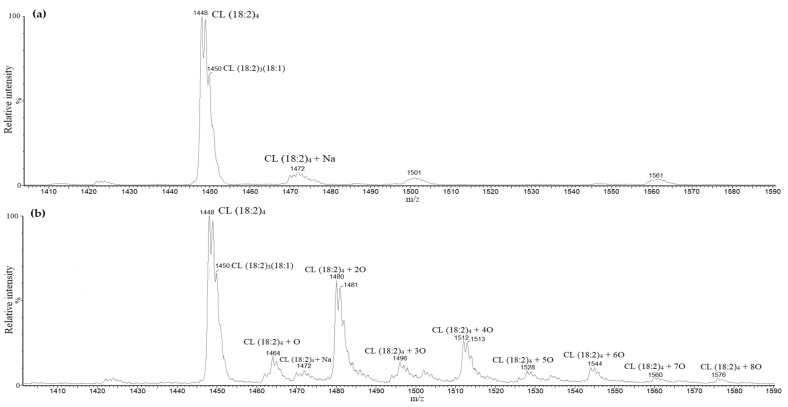
Cardiolipin standard mass spectra before (**a**) and after oxidation (**b**). After oxidation of cardiolipin standard, masses of tetralinoleoyl cardiolipin with an additional one to eight oxygen atoms were observed.

To determine possible CL oxidation products that could be formed during kidney ischemia in vivo, oxidation of CL standard using cytochrome c with H_2_O_2_ was performed. Several oxidized CL forms with up to eight extra oxygen atoms were determined. Figure 3 shows mass spectra differences before (a) and after oxidation (b) of the cardiolipin standard. The more extra oxygen atoms CL had, the more hydrophilic it became and the earlier it was eluted from the chromatographic column. Additionally, the oxidation of the CL standard yielded various distributions of oxygen atoms on the linoleic fatty acids, which created different oxidated CL isomers. Some of the oxidated fatty acids became dehydrated due to the loss of one water molecule (m/z − 18 after oxidation), which is typical for lipid hydroperoxides.

**Figure 4:**
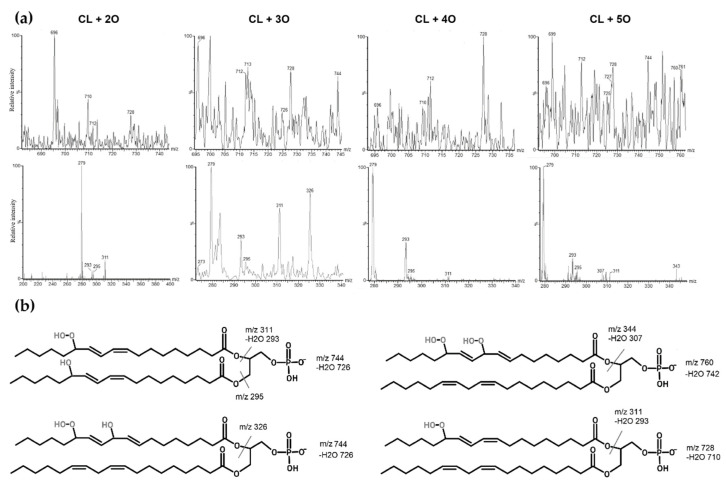
Mass spectra of oxidized tetralinoleoyl cardiolipin with an additional 2, 3, 4 and 5 oxygen atoms. (**a**) The oxidized phosphatidic acid fragments are shown in top mass spectra, and the oxidized linoleic acid fragments are shown in bottom mass spectra. (**b**) Several possibilities of oxidized tetralinoleoyl phosphatidic acid fragment structures are presented as well.

CL with one extra oxygen atom (m/z 1464) was eluted and identified at 15.21 min. Mass spectra of the peak at 15.21 showed fragments with m/z 279 corresponding to linoleic acid, m/z 295 corresponding to linoleic acid hydroxide (m/z 279 + 16) and m/z 712 corresponding to phosphatidic acid hydroxide (m/z 696 + 16). At 13.94 min, an oxidized CL with two extra oxygen atoms attached to the molecule (m/z 1480) was observed. Mass spectra showed m/z 311 corresponding to hydroperoxide (m/z 279 + 32) and m/z 293 corresponding to linoleic acid monohydroperoxide, which was dehydrated (m/z 311 − 18). Additionally, m/z 728 corresponding to phosphatidic acid hydroperoxide (m/z 696 + 32) and m/z 710 corresponding to phosphatidic acid hydroperoxide were observed with loss of water (m/z 728 − 18). There was another m/z 1392, which corresponded to CL hydroperoxide with the loss of a fragment of five carbons and oxygen, indicating that the oxidation occurred at the 13th carbon of the linoleic acid. The mass spectra of this chromatographic peak also included m/z 1464, 295 and 712, which correspond to monohydroxylated cardiolipin, possibly showing a reduction of monohydroperoxide. However, m/z 712 and 295 point to a dihydroxylated cardiolipin. Cardiolipin oxidized with three extra oxygen atoms (m/z 1496) was eluted at 12.20 min. The mass spectrum of this species showed a prominent fragment of m/z 326, which points to linoleic acid with hydroxyl and hydroperoxyl groups. Fragments m/z 744 show phosphatidic acid with three (m/z 696 + 48) additional oxygens, respectively. A minor fragment of m/z 726 might indicate a dehydrated phosphatidic acid hydroperoxide with a hydroxy group (m/z 744 − 18). Some fragments with less intensity were m/z 293, 295 and 311, and these showed linoleic acid hydroxides and hydroperoxides. Cardiolipin oxidized with four additional oxygen atoms (m/z 1512) was eluted at 10.18 min. The mass spectrum of this chromatographic peak showed prominent fragments of m/z 293 and m/z 311, showing linoleic acid with two oxygens (m/z 279 + 32), which is a dehydrated hydroperoxide (m/z 311 − 18). Fragments of m/z 712 and 728 show phosphatidic acids with one and two additional oxygens, respectively. A larger fragment of m/z 1424 was observed, possibly indicating a loss of 5-carbon and oxygen fragments, again pointing to peroxidation at the 13th carbon of linoleic acid. These findings might show different isomeric forms, including both hydroperoxides and hydroxides of this oxidized CL species. Cardiolipin with five additional oxygen atoms (m/z 1528) was eluted at 8.87. Mass spectra showed fragments of m/z 293, 295, 311 showing hydroxy and hydroperoxy linoleic acids. Larger fragments of m/z 728 and 744 show phosphatidic acids with two and three additional oxygens. Additionally, smaller fragments of m/z 344 and 307 were observed, which could correspond to a linoleic acid dihydroperoxide with dehydration. Another fragment was present but not prominent—m/z 760, a phosphatidic acid with four additional oxygens. Cardiolipin with an additional six oxygen atoms (m/z 1544) was eluted at 6.80 min. Most of the observed fragments in the mass spectrum were of linoleic acid oxides—m/z 293, 295, 311, 327 and 344. All of these correspond to hydroxides, hydroperoxides, and dihydroperoxide, as mentioned before. Additionally, mass spectra presented other masses, including m/z 1528 and 1512, which show the reduction of the oxidized m/z 1544 form. Next, cardiolipin with an additional seven oxygen atoms with m/z 1560 was observed at 5.30 min. Mass spectra showed similar fatty acid oxides with more prominent m/z 293 and 311 showing hydroperoxides. Larger fragments of m/z 712 and 728 were also present, as well as m/z 742, which might show a dehydrated phosphatidic acid with a hydroperoxy group (m/z 760 − 18). Cardiolipin with eight extra oxygen atoms was eluted at around 4.30 min. Mass spectra showed m/z 293 and 311 of linoleic acid hydroperoxides. Furthermore, m/z 295 linoleic acid hydroxide and m/z 328 linoleic acid with hydroxy and hydroperoxy groups were also shown. Larger fragments included m/z 712, 728 and even m/z 760 and 742 (m/z 760 − 18), which might point to a phosphatidic acid bishydroperoxide. Another fragment with m/z 1488 was shown, which might point to a loss of a fragment with five carbons and oxygen, as mentioned before. The mass spectra of the last four oxidized cardiolipin forms (especially of m/z 1560 and m/z 1576) demonstrated a low signal-to-noise ratio; therefore, there were many nonspecific mass signals that might have interfered with the overall interpretation of these oxidation forms. These results present an array of tetralinoleoyl cardiolipin oxides with a variety of oxidized linoleic acid forms. Several of the possible forms are presented in Figure 4. Overall, the highest relative signal intensity was observed with cardiolipin that was oxidized with additional two oxygen atoms, and the lowest was with cardiolipin with an additional seven and eight oxygen atoms. The retention factors for each of these oxygenated cardiolipin forms were calculated (the ratio of the retention times of oxidized cardiolipin isoforms to the retention times of the non-oxidized CL). These retention factors, along with mass spectra, were used to locate the chromatographic peaks of oxidized cardiolipin forms in the biological samples.

### 3.3. Identification of Cardiolipin in Rat Kidney Mitochondria

**Figure 5:**
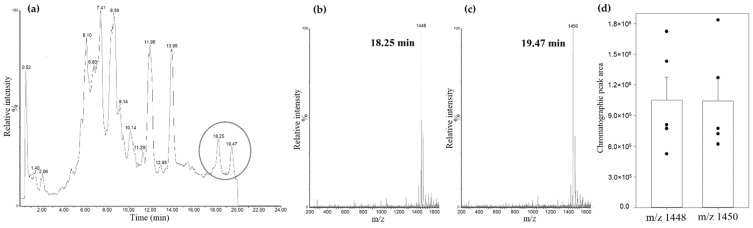
Rat kidney mitochondrial lipid fraction UPLC-MS analysis. (**a**) total scan chromatogram (negatively charged lipids); (**b**) mass spectrum at 18.25 min retention time; (**c**) mass spectrum at 19.49 min retention time; (**d**) comparison of cardiolipins’ m/z 1448 and m/z 1450 chromatographic peak areas, (*n* = 5, dots represent values of each experiment, means ± standard error).

Following the analysis of the CL standard and its oxidation, an analysis of the control rats’ kidney mitochondrial lipid fractions was performed. Since cardiolipin constitutes only 2–3% [31] of total kidney lipids, the chromatographic peaks of cardiolipin were not prominent among other negatively charged phospholipids in the used chromatographic conditions (Figure 5a). However, mass spectrometry analysis confirmed cardiolipin species with similar chromatographic retention times as observed during the chromatographic analysis of the cardiolipin standard. Two cardiolipin species that were present in the rat kidney mitochondrial lipid fraction were observed: cardiolipin with four linoleic fatty acids (m/z 1448) and cardiolipin with three linoleic and one oleic fatty acid (m/z 1450) (Figure 5b,c). The peak areas of these cardiolipin species were integrated, and it was discovered that they were quite similar, indicating that the amounts of these cardiolipin species in rat kidney mitochondria are the same (Figure 5d).

### 3.4. Analysis of Cardiolipin Oxidation in Rat Kidney Mitochondria during Kidney Ischemia In Vivo

**Figure 6:**
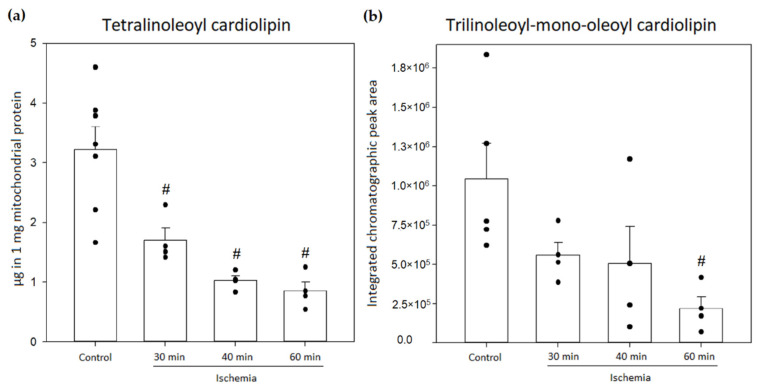
Changes in amounts of mitochondrial cardiolipin in the control group and after 30, 40 and 60 min of kidney ischemia. (**a**) Changes in the amounts of tetralinoleoyl cardiolipin (*n* = 4–7); (**b**) changes in the relative amounts of trilinoleoyl-mono-oleoyl cardiolipin (*n* = 4–5). (# *p* < 0.05 control vs. ischemia, dots represent values of each experiment, mean ± standard error).

In the next step, cardiolipin oxidation in rat kidney mitochondria after warm kidney ischemia (30–60 min) in vivo was investigated. Firstly, the amount of cardiolipin in rat kidney mitochondria was measured after performing warm kidney ischemia, and it was found that after 30 min, 40 min and 60 min of ischemia, tetralinoleoyl cardiolipin significantly decreased by 47%, 68% and 74%, respectively, compared to pre-ischemic amounts of tetralinoleoyl cardiolipin (*p* < 0.05, Welch’s *t*-test, Figure 6a). The changes in the amount of trilinoleoyl-mono-oleoyl cardiolipin were determined by comparing the integrated chromatographic peak area values that were adjusted to 1 mg mitochondrial protein. The results showed that after 30 min of ischemia, the peak area of trilinoleoyl-mono-oleoyl cardiolipin decreased by 46%; however, 60 min of ischemia caused a significant decrease of 79% (*p* < 0.05, Student’s *t*-test, Figure 6b). This shows that both cardiolipin species in the kidneys are consumed during ischemia; however, the oxidation of the major tetralinoleoyl cardiolipin species was investigated in further analysis.

**Figure 7:**
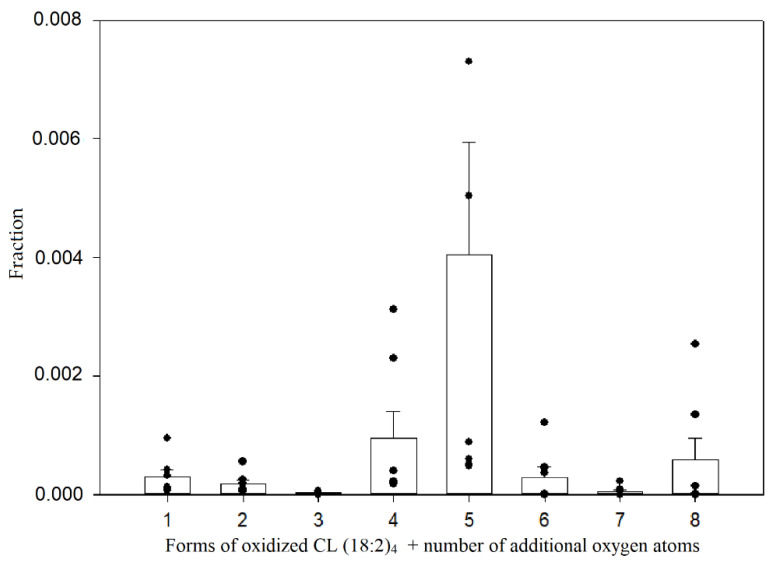
Relative amounts of oxidized tetralinoleoyl cardiolipin species in control mitochondria. The relative quantification was computed by calculating the fraction of the integrated peak area by the total peak area of all observed oxidized and non-oxidized tetralinoleoyl cardiolipin species; (*n* = 7, dots represent values of each experiment, mean ± standard error).

By using retention factors that were mentioned earlier, approximate retention times for oxidized cardiolipin forms were calculated and used to discover that small amounts of these forms can be detected even in control mitochondria (pre-ischemic kidneys). Among these oxidized cardiolipin species, the dominant ones were oxidized with an additional four and five oxygen atoms (Figure 7). 

**Figure 8:**
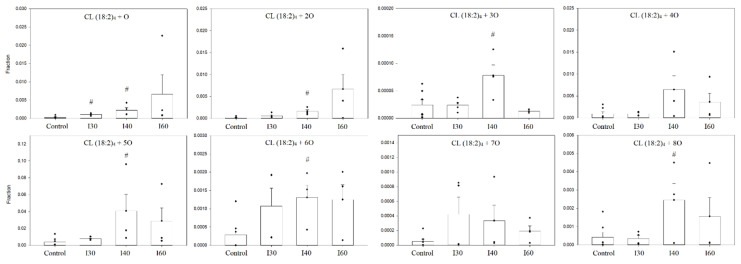
Relative amounts of oxidized cardiolipin species of control and ischemic kidney mitochondria. There was a significant increase of most oxidized cardiolipin species after 40 min ischemia. The relative quantification was computed by calculating the fraction of the integrated peak area by total peak are of all observed oxidized and non-oxidized tetralinoleoyl cardiolipin species. I 30—30-min ischemia, I40—40-min ischemia, I60—60-min ischemia; CL (18:2)_4_ + number of additional oxygen atoms; *n* = 4–7; (# *p* < 0.05 control vs. ischemia, dots represent values of each experiment, mean ± standard error).

These two species were predominant in all ischemic groups as well. To compare the amounts of these oxidized species, a relative quantification was performed, which was based on the fractions of integrated peak areas of these oxidized forms with the total peak area that was a sum of all oxidized species, including the non-oxidized tetralinoleoyl cardiolipin. The changes in the relative amounts of different oxidized cardiolipin molecular forms are presented in Figure 8.

An average 6.9-fold increase in all oxidized cardiolipin forms after 40 min ischemia was observed. A great increase was observed with monooxygenated tetralinoleoyl cardiolipin CL (18:2)_4_ + 1O, which was observed at 30 min of ischemia—the relative amount of this species significantly increased 3.72-fold, and after 40 min of ischemia, the increase was 8.79-fold compared to control (*p* < 0.05, Student’s *t*-test). However, after 60 min of ischemia, the relative amount of CL (18:2)_4_ + 1O was already 27.15 times higher than the control levels. Similar changes were observed analyzing relative amounts of CL (18:2)_4_ + 2O oxidized species. After 40 min of ischemia, the relative amount of this species increased 9.09-fold compared to control (*p* < 0.05, Student’s *t*-test), and after 60 min, it was 37.84 times higher than control levels. After 40 min of ischemia, the relative amounts of oxidized cardiolipin species with an additional three, five, six and eight oxygen atoms increased 3.25-, 10.17-, 4.50- and 5.88-fold, respectively, compared to control levels (*p* < 0.05, Student’s *t*-test (Mann–Whitney sum rank test for CL (18:2)_4_ + 5O)). However, after 60 min of ischemia, there was a tendency to see a decrease in the relative amount of these species, including CL (18:2)_4_ + 4O and CL (18:2)_4_ + 7O, compared to 40 min of ischemia.

## 4. Discussion

Ischemia and reperfusion remain a serious clinical problem that damages the affected organs and tissues. The molecular mechanism of this injury involves increased reactive oxygen species generation in mitochondria and cardiolipin peroxidation and depletion, which in turn impairs mitochondrial oxidative phosphorylation and mitochondrial and cellular integrity [21,32,33]. Mitochondrial damage already occurs during ischemia, and although the level of oxygen is reduced, reactive oxygen species are still generated at a level that is enough to oxidize and deplete cardiolipin [34]. Cardiolipin peroxidation during ischemia-reperfusion has mostly been analyzed in heart mitochondria; however, there is a lack of research that has been done on cardiolipin in renal mitochondria. 

This study shows for the first time that tetralinoleoyl cardiolipin is oxidatively modified and depleted in rat kidney mitochondria during in vivo kidney ischemia. In the first stage, a standard tetralinoleoyl cardiolipin was used for in vitro oxidation using cytochrome c and hydrogen peroxide for the analysis of cardiolipin oxidation products. Since cardiolipin closely interacts with cytochrome c in the mitochondria, cardiolipin can be readily oxidized by cytochrome c peroxidase [35]. Additionally, Aluri et al. showed that during mouse heart ischemia and reperfusion, the inhibition of the distal part of the electron transport chain, mainly the IV complex, evokes cytochrome c peroxidase activity, which, together with hydrogen peroxide, can readily oxidize cardiolipin [36]. Using UPLC-MS, eight cardiolipin oxidation products that had from one to eight additional oxygen atoms attached to linoleic acids were evaluated. Based on mass spectrometry results, up to four oxygen atoms could be found on a single linoleic acid in the forms of hydroxides or hydroperoxides. Similar findings were also published by Kim et al.; however, different oxidizing agents were used, specifically air/2,2’-azobis-2-methyl-propanimidamide,dihydrochloride (AAPH)/photosensitizer and light. Furthermore, similar findings were also published by Helmer et al., who applied a Fenton reaction. The authors described in detail cardiolipin oxidation products with additional two and four oxygen atoms. Even though Helmer et al. showed that up to eight oxygen atoms were detected on cardiolipin, no further details on the rest of the cardiolipin forms were provided [30,37]. The ion fragmentation patterns were similar to those described in this study, which may show that cardiolipin oxidation with cytochrome c and hydrogen peroxide follow the same patterns of oxidation. Among the oxidation products of standard cardiolipin, the dominating form was cardiolipin with two additional oxygen atoms—cardiolipin hydroperoxide, which was also observed by Helmer et al. [37]. However, our study shows that in the in vivo setting, the relative amounts of oxidized cardiolipin forms might differ since the most abundant forms were cardiolipin with an additional five and four oxygen atoms in control and ischemic rat kidney mitochondria. This indicates that cardiolipin hydroperoxide might still be reactive and undergo further oxidation in vivo, producing somewhat more stable oxidized cardiolipin forms. 

In the next set of experiments, we analyzed the quantitative and qualitative changes of rat kidney cardiolipin after 30–60 min in vivo ischemia. Firstly, it was determined that the levels of tetralinoleoyl cardiolipin and trilinoleoyl-monooleoyl cardiolipin are almost the same in rat kidney mitochondria, which makes both of them dominant. After 30 min of in vivo ischemia, the levels of both cardiolipin species decrease in half; however, only after 30 min and 40 min ischemia is the decrease significant for tetralinoleoyl cardiolipin. Petrosillo et al. analyzed cardiolipin peroxidation in ischemic rat heart and demonstrated that 30 min of ischemia caused a decrease in cardiolipin content by 28% [33]. Similar results were obtained by Lesnefsky et al. while analyzing rabbit heart mitochondria in ischemic conditions. A total of 30 min ischemia caused a slight decrease in cardiolipin and cytochrome c content in subsarcolemmal mitochondria; however, no change in cardiolipin or cytochrome c content was observed in interfibrillar mitochondria [25]. Chen and Lesnefsky further investigated this model of ischemia and found that after 30 and 45 min of ischemia, the production of hydrogen peroxide significantly increased, which means that cardiolipin can be readily oxidated in ischemic conditions [38]. It has also been shown that even 20 min of warm renal ischemia in vitro caused significant damage to the mitochondrial oxidative phosphorylation system as well as an increase in proapoptotic factor activity [39]. However, these experiments were done ex vivo/in vitro, and so far, not many in vivo experiments have been done to provide any information about cardiolipin alterations during ischemia alone, especially in kidneys. Kim et al. analyzed cardiolipin content in rat brain and heart after asphyxia-induced cardiac arrest and discovered that tetralinoleoyl cardiolipin is only slightly depleted in rat heart, but not the brain, after 30 min of cardiac arrest [40]. Liu et al. induced 45 min of rat kidney ischemia in vivo and discovered that kidney capillary endothelial cell mitochondria became swollen with a loss of cristae membranes which was attenuated by a cardiolipin-targeted peptide SS-31. Even though the authors did not analyze cardiolipin, their results indicate that the loss of cardiolipin could have induced morphological changes in mitochondria [41]. 

Not much is known about cardiolipin oxidation products during ischemia, especially in ischemia in vivo. This study shows that rat kidney ischemia in vivo causes tetralinoleoyl cardiolipin oxidation with up to eight additional oxygen atoms, forming eight oxidation products. It was shown, by Lesnefsky et al., that 25 min of aged rat heart ischemia caused a significant increase in 1496 Dalton cardiolipin (with an additional three oxygen atoms) in subsarcolemmal and interfibrillar mitochondria. Interestingly, the amount of this cardiolipin species in rat heart mitochondria after 25 min of ischemia was higher compared to 25 min of ischemia followed by 30 min of reperfusion [26]. Our study showed that this oxidized cardiolipin species significantly increases after 30 min of in vivo rat kidney ischemia; however, it decreases almost to control levels after 60 min of ischemia. Such a tendency was noticed with other cardiolipin oxides that had an extra four, five, six, seven and eight oxygen atoms. We hypothesize that longer ischemia times might trigger oxidized cardiolipin hydrolysis by phospholipases into various lysocardiolipin species and linoleic acid peroxides [42,43]. Indeed, Smaalen et al. have used a porcine model of clinical kidney transplantation design with subnormothermic (28 °C) ischemic perfusion and showed that kidney ischemia in pigs causes significant elevation of monolysocardiolipin (trilinoleoyl cardiolipin) [44]. Such monolysocardiolipins are no longer suitable for the induction of mitochondrial inner membrane curvature, nor do they associate with mitochondrial proteins or native cardiolipin, which causes a decrease in the activity of oxidative phosphorylation and release of cytochrome c [45]. Paradies et al. have shown that loss of cardiolipin caused a decrease in the activity of complexes I, III and IV, which was restored with exogenous cardiolipin, but not with monolysocardiolipin or oxidated cardiolipin [11,12,13]. 

## 5. Conclusions

This study shows that kidney ischemia in vivo alone causes severe cardiolipin oxidation and depletion in rat kidney mitochondria. Further studies are needed to assess possible metabolites (like monolysocardiolipin, free linoleic acid peroxides, and 4-hydroxynonenal) that can come out of the catabolism of oxidized cardiolipin and participate in cell signaling that may lead to renal cell death. Additionally, the prevention of cardiolipin oxidation and depletion during ischemia could protect kidneys from the increased damage that occurs during reperfusion.

## Figures and Tables

**Table 1:** Elution gradient for cardiolipin separation and analysis with UPLC.

Time (min)	A Eluent (vol%)	B Eluent (vol%)	Flow Rate (mL/min)
Initial	100	0	0.5
10	50	50	0.5
20	20	80	0.35
21	0	100	0.2
24	100	0	0.2
26	100	0	0.5

## Data Availability

The data that support the findings of this study are available from the corresponding author upon request.
